# Translating polygenic risk scores for clinical use by estimating the confidence bounds of risk prediction

**DOI:** 10.1038/s41467-021-25014-7

**Published:** 2021-09-06

**Authors:** Jiangming Sun, Yunpeng Wang, Lasse Folkersen, Yan Borné, Inge Amlien, Alfonso Buil, Marju Orho-Melander, Anders D. Børglum, David M. Hougaard, Regeneron Genetics Center, Luca Andrea Lotta, Marcus Jones, Aris Baras, Olle Melander, Gunnar Engström, Thomas Werge, Kasper Lage

**Affiliations:** 1 Institute of Biological Psychiatry, Mental Health Center Sct. Hans, Mental Health Services Copenhagen, Roskilde, Denmark; 2 The Lundbeck Foundation Initiative for Integrative Psychiatric Research (iPSYCH), Copenhagen, Denmark; 3 Department of Clinical Sciences, Malmö, Lund University, Malmö, Sweden; 4 Lifespan Changes in Bain and Cognition (LCBC), Department of Psychology, University of Oslo, Oslo, Norway; 5 Department of Biomedicine, Human Genetics and Centre for Integrative Sequencing, Aarhus University, Aarhus, Denmark; 6 Department for Congenital Disorders, Center for Neonatal Screening, Statens Serum Institut, Copenhagen, Denmark; 7 Regeneron Genetics Center, Regeneron Pharmaceuticals, Tarrytown, NY USA; 8 Department of Clinical Medicine, Faculty of Health and Medical Sciences, University of Copenhagen, Copenhagen, Denmark; 9 Stanley Center at Broad Institute of MIT and Harvard, Cambridge, MA USA; 10 Department of Surgery, Massachusetts General Hospital, Boston, MA USA

**Keywords:** Machine learning, Population genetics, Predictive markers

## Abstract

A promise of genomics in precision medicine is to provide individualized genetic risk predictions. Polygenic risk scores (PRS), computed by aggregating effects from many genomic variants, have been developed as a useful tool in complex disease research. However, the application of PRS as a tool for predicting an individual’s disease susceptibility in a clinical setting is challenging because PRS typically provide a relative measure of risk evaluated at the level of a group of people but not at individual level. Here, we introduce a machine-learning technique, Mondrian Cross-Conformal Prediction (MCCP), to estimate the confidence bounds of PRS-to-disease-risk prediction. MCCP can report disease status conditional probability value for each individual and give a prediction at a desired error level. Moreover, with a user-defined prediction error rate, MCCP can estimate the proportion of sample (coverage) with a correct prediction.

## Abstract (web summary)

The application of polygenic risk scores to individual-level disease susceptibility is challenging, as risk is evaluated at a group-level. Here, the authors describe a machine learning method, Mondrian Cross-Conformal Prediction, that reports disease status conditional probability value at the individual level.

## Introduction

The last decade has witnessed the tremendous success of genome-wide association studies (GWAS), which have discovered tens of thousands of common variants robustly associated with a range of human complex traits and diseases^1^, including cancers^2,3^, cardiovascular diseases^4^, neuropsychiatric, and neurodegenerative diseases^5–7^. Although the identified variants individually have small to modest effect sizes, polygenic risk scores (PRS)^8,9^, which summarize effects from large numbers of variants, have proven to be a useful research tool. For example, PRS has been used to investigate the genetic overlaps of neuropsychiatric disorders^8,10–12^, identify individuals of high risk for coronary artery disease^13^, predict the age of onset for Alzheimer’s disease^14^, and improve clinical diagnoses of cancers^15,16^ and type 2 diabetes mellitus^17^. Compared with environmental risk factors, PRS has many advantages. As an individual’s DNA is largely stable after conception, PRS for complex disorders is also stable. Therefore, it is unlikely that non-genetic factors can cause large numbers of changes to DNA, i.e., inverse causations. In addition, PRS is easy to compute. Thus, there is little doubt that the implementation of PRS will be an integral part of the field of precision medicine^18–20^.

However, there are several technical obstacles to using PRS in clinical settings. In contrast to rare disease-causing mutations, which have large penetrance, PRS are continuous measures of the liability to disease as well as probabilistic measures of the risk of developing a condition^21,22^. Thus, it is unclear what thresholds of PRS should be used by clinicians to assess an individual’s risk to develop a disease. To mitigate this, standard statistical models in the field divide the sample into different strata based on arbitrary PRS thresholds and evaluate the effect of PRS on disease risk within and across strata by several statistical metrics, for example, area under the receiver operating curve (AUC), the proportion of risk variation explained (R^2^), odds ratio, and hazard ratio. The deciles, quintiles, top 10%, 5%, and even 1% versus bottom 10%, 5%, and 1%, respectively, are frequently used thresholds in the literature^5,10,14–16^. As the performance of PRS is intimately related to genetic architectures of complex disorders^22^, which vary for different disorders and in different populations, a systematic strategy for choosing these risk stratifying thresholds is imperative. In addition, the prediction accuracy per individual has rarely been investigated in PRS studies.

**Fig. 1:**
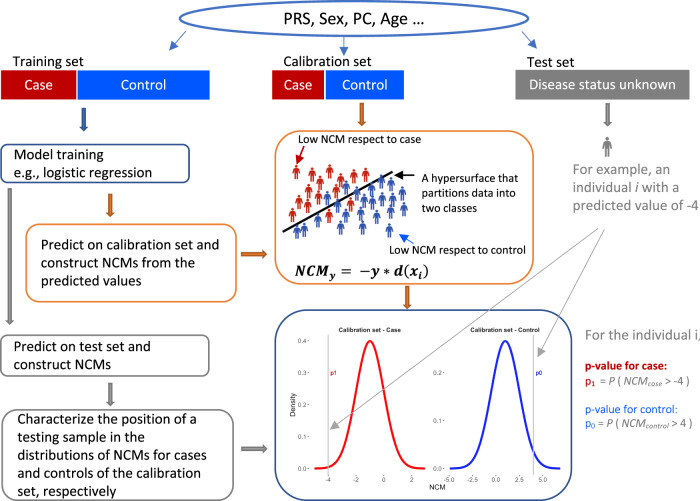
Schema of the MCCP for the PRS-to-trait prediction. Using summary statistics from a reference GWAS, PRS is obtained for the target population. The calibration set includes individuals with known disease status. A model is built on the training set and nonconformity measures (NCM) can be computed for both calibration and test sets, respectively. The NCMs distributions in the calibration set are obtained for case and control, respectively (blue and red curves). The MCCP probability values for an individual to be the case (*p*_1_) or control (*p*_0_) (gray vertical bar) are estimated, respectively. In principle, population structures, age, sex, and other covariates can also be added to the model to increase its performance.

Here, we introduce a machine-learning technique, Mondrian Cross-Conformal predictor (MCCP), to complement the current state-of-the-art PRS methodology. In contrast to arbitrary PRS thresholds used in the literature, MCCP, functioning as a calibrator (Fig. 1) for PRS prediction in a test sample, is able to compute the proportion of the sample (termed coverage hereafter) for which the prediction of case-control status is reliable, i.e., below a pre-specified prediction error rate. For an individual with a predicted status, MCCP can estimate the confidence bound of the prediction. We evaluated the performance of MCPP on a range of simulated genetic architectures that are frequently observed in empirical studies. We applied MCCP on coronary artery disease (CAD), type 2 diabetes mellitus (T2D), inflammatory bowel disease (IBD), and breast cancer (BRCA) using the UK Biobank resource^23,24^ and on two additional population-based data sets, the Integrative Psychiatric Research (iPSYCH) schizophrenia (SCZ)^25^ sample and the Malmö Diet and Cancer (MDC) T2D^26^ sample.

Overall, we show that at the individual level, MCCP reports well-calibrated prediction probabilities, systematically estimates confidence bounds of PRS-to-risk prediction of human complex diseases. At the group level, MCCP outperforms standard methods in accurately stratifying individuals into risk groups.

## Results

### Overview of the method

MCCP is a special implementation of conformal prediction (CP) in classification that can guarantee the validity of the conformal predictor for each class (here, case and control separately)^27,28^. MCCP splits the sample by their respective classes and then estimates confidence levels for each class. Here, we implemented MCCP to estimate the confidence levels of risk prediction in a sample for which genetic and disease status information was available (Fig. 1; target sample). Our implementation first computed PRS for each individual in the target sample. We, then, divided the target sample into two subsets: the training and the testing set. The training set was further randomly partitioned into *n*equal-sized subsets, one of which was retained as the calibration subset for calculating the MCCP probability value described by Eq. (1), and the remaining *n*−1 subset was used as the proper training set for model building. We fitted a logistic regression model on the proper training set and made predictions on both the calibration and the testing sets. A nonconformity measure (NCM; Fig. 1 and Methods) was calculated for every individual in the calibration and testing sets. Assuming that the training and the calibration sets were independent and identically distributed, we ranked the NCMs in the calibration set for both the case and control groups, respectively. Based on NCMs in the calibration set, probability values for assigning case or control labels to reach individuals in the testing set were then computed (equation [1]). We repeated this procedure *n* times, using each of the *n* subsamples exactly once as the calibration set. The *n* probability values were averaged to produce a single estimation for the final prediction region of predicted subjects.

1 $${p}_{y}^{i}=\frac{|\{j=1,\,\ldots ,N\_{{{{{\rm{cal}}}}}}_{y}:\,{y}_{i}=y,\,{{{{{\rm{NCM}}}}}}_{j}\ge {{{{{\rm{NCM}}}}}}_{i}\}|}{(N\_{{{{{\rm{cal}}}}}}_{y}+1:{y}_{i}=y)}$$

where${p}_{y}^{i}$ is the probability value of individual *i* to be in class *y* and *N_*cal_*y*_ is the sample size of class *y* in the calibration set.

In binary classifications, the probability values for assigning an individual as a case (*p*_1_) and a control (*p*_0_) are obtained from the MCCP, respectively. Given a prediction error rate of *α*, a subject can be predicted by MCCP as a case (*p*_1_ > *α* and *p*_0_ ≤ *α*) or a control (*p*_0_ > *α* and *p*_1_ ≤ α), uncertain (*p*_1_ > *α* and *p*_0_ > α) or unpredictable (*p*_1_ ≤ α and *p*_0_ ≤ *α*) with a confidence level of 1−*α*.

The prediction coverage is defined as the proportion of samples predicted as case or control at the given error rate *α*. To assess the clinical significance of the MCCP results versus standard methods, we computed the AUC, positive predictive value (PPV), and negative predictive value (NPV) restricted to predicted cases and controls at an error rate α.

### Prediction error and coverage of MCCP

**Fig. 2:**
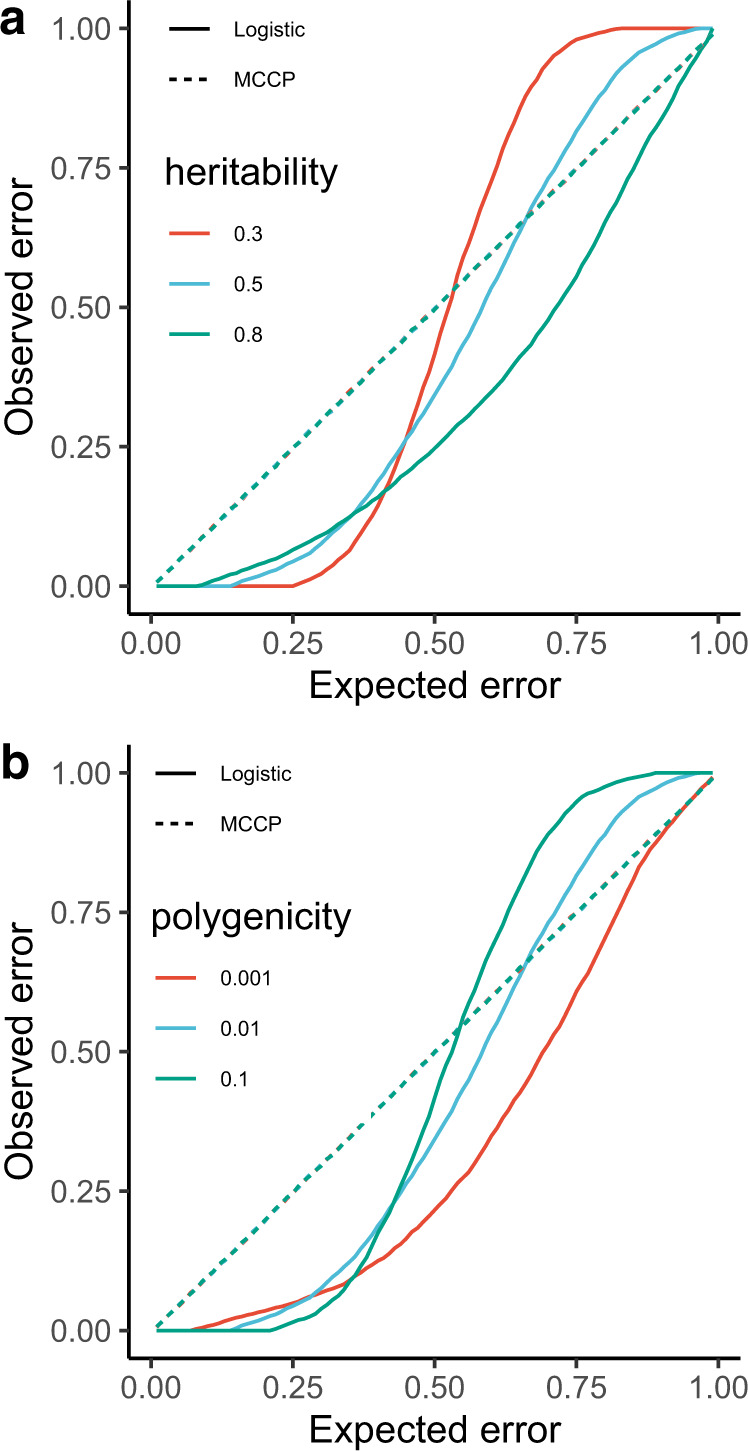
Calibration plots comparing observed and expected errors from MCCP and the logistic regression model using PRS as a predictor on the simulated data. **a** Curves for the prevalence of 0.01, polygenicity of 0.01, and heritability (h^2﻿^) of 0.3, 0.5, and 0.8 are shown. **b** Curves for the prevalence of 0.01, the heritability of 0.5, and polygenicity of 0.001, 0.01, and 0.1 are shown. The logistic regression is fitted on the combined proper training and calibration sets and used to make predictions on the test set. The observed error is measured as the proportion of incorrect predictions against true cases or controls status at an expected error rate using the estimated MCCP probability values and probabilities from the logistic regression, respectively. Optimal calibration is shown as dashed gray line that is largely overlapped with MCCP curves. Source data are provided as a Source Data file.

We examined the calibration property of MCCP on prediction error for PRS across a range of simulated genetic architectures and case-control GWAS designs based on real genotypes (Methods and Supplementary Figure 1). For each simulated data set, MCCP and a simple logistic regression (LR) model were applied to the proper training and calibration sets. The predicted errors for the two models were compared in the hold-off testing set (Methods). In line with previous studies on CP^27,29^, the prediction errors from MCCP were perfectly aligned with those expected across all simulated scenarios (Fig. 2 and Supplementary Figures 1–4). However, the naively implemented LR model underestimated the error rate when it was small; but, tended to overestimate when it was large (Fig. 2). Moreover, such biases varied with different genetic architectures. For example, for a fixed polygenicity of 0.01 and prevalence of 0.01, the LR models consistently underestimated the true error rate for heritability of 0.8, and, for a fixed heritability of 0.5 when the true error rate was < 0.5. A similar finding was found for cases with a low polygenicity (i.e., 0.001). These results suggested that MCCP was superior in calibrating PRS prediction compared with the naive LR model.

Next, we studied the coverage of MCCP—how many subjects can be predicted at a given error rate—in our simulated data set (Supplementary Fig. 5). We observed that, as expected, the maximum coverage was achieved with a low error rate when the heritability was high. This was also true for data sets with low polygenicity for a fixed prevalence and heritability. Low prevalence tended to achieve high coverage at a lower error rate compared with high prevalence. This was mainly caused by the inherent imbalance of case versus control in the data. Moreover, as expected, larger GWAS discovery studies, which can generate more accurate effect estimates than smaller ones, always showed better prediction performance. Performance of MCCP using fivefold cross-validation at an error rate of 0.05 is given in Table 1.

**Table 1:** Performances of Mondrian cross-conformal prediction at an error of 0.05 on various genetic architecture by simulation (proportion of discovery set for GWAS is 0.5).

Heritability	Polygenicity	Prevalence	AUC (95% CI)	PPV (95% CI)	NPV (95% CI)	Coverage
0.3	0.001	0.01	0.84 (0.80–0.88)	0.48 (0.44–0.53)	0.96 (0.94–0.98)	0.18
		0.05	0.81 (0.79–0.84)	0.76 (0.73–0.80)	0.81 (0.77–0.84)	0.22
		0.2	0.76 (0.73–0.79)	0.75 (0.72–0.81)	0.67 (0.61–0.72)	0.17
	0.01	0.01	0.78 (0.71–0.84)	0.25 (0.19–0.35)	0.97 (0.95–0.99)	0.16
		0.05	0.75 (0.71–0.79)	0.61 (0.57–0.65)	0.82 (0.79–0.85)	0.18
		0.2	0.73 (0.70–0.77)	0.69 (0.66–0.73)	0.74 (0.71–0.78)	0.17
	0.1	0.01	0.67 (0.58–0.76)	0.18 (0.13–0.33)	0.95 (0.92–0.99)	0.12
		0.05	0.67 (0.62–0.71)	0.57 (0.52–0.63)	0.74 (0.70–0.78)	0.14
		0.2	0.59 (0.55–0.64)	0.65 (0.59–0.73)	0.56 (0.53–0.61)	0.12
	0.2	0.01	0.68 (0.60–0.76)	0.24 (0.20–0.29)	0.94 (0.91–0.97)	0.11
		0.05	0.70 (0.65–0.74)	0.61 (0.57–0.65)	0.76 (0.72–0.80)	0.15
		0.2	0.62 (0.58–0.67)	0.65 (0.60–0.69)	0.62 (0.58–0.66)	0.16
0.5	0.001	0.01	0.91 (0.89–0.94)	0.57 (0.52–0.61)	0.98 (0.97–0.99)	0.32
		0.05	0.88 (0.86–0.90)	0.81 (0.79–0.84)	0.88 (0.86–0.90)	0.30
		0.2	0.83 (0.80–0.85)	0.82 (0.79–0.84)	0.78 (0.75–0.81)	0.24
	0.01	0.01	0.85 (0.81–0.90)	0.39 (0.34–0.44)	0.98 (0.96–0.99)	0.23
		0.05	0.84 (0.82–0.87)	0.72 (0.69–0.76)	0.88 (0.86–0.90)	0.26
		0.2	0.80 (0.77–0.82)	0.77 (0.74–0.80)	0.79 (0.76–0.82)	0.21
	0.1	0.01	0.75 (0.69–0.81)	0.28 (0.24–0.33)	0.96 (0.94–0.98)	0.15
		0.05	0.80 (0.77–0.83)	0.69 (0.66–0.73)	0.83 (0.81–0.86)	0.21
		0.2	0.70 (0.66–0.73)	0.68 (0.64–0.72)	0.71 (0.67–0.74)	0.15
	0.2	0.01	0.79 (0.73–0.85)	0.20 (0.16–0.25)	0.98 (0.96–1.00)	0.17
		0.05	0.75 (0.72–0.79)	0.64 (0.60–0.69)	0.83 (0.80–0.86)	0.19
		0.2	0.68 (0.64–0.72)	0.65 (0.61–0.70)	0.68 (0.64–0.72)	0.14
0.8	0.001	0.01	0.93 (0.90–0.95)	0.50 (0.43–0.55)	0.99 (0.98–1.00)	0.42
		0.05	0.92 (0.90–0.93)	0.82 (0.79–0.85)	0.94 (0.93–0.95)	0.46
		0.2	0.91 (0.90–0.92)	0.88 (0.87–0.90)	0.87 (0.85–0.89)	0.39
	0.01	0.01	0.91 (0.88–0.93)	0.50 (0.46–0.55)	0.98 (0.97–0.99)	0.31
		0.05	0.91 (0.90–0.93)	0.83 (0.80–0.85)	0.93 (0.91–0.94)	0.41
		0.2	0.89 (0.87–0.90)	0.85 (0.82–0.87)	0.86 (0.84–0.88)	0.33
	0.1	0.01	0.84 (0.79–0.89)	0.36 (0.31–0.42)	0.98 (0.96–0.99)	0.21
		0.05	0.86 (0.83–0.88)	0.75 (0.72–0.78)	0.89 (0.86–0.91)	0.28
		0.2	0.82 (0.79–0.84)	0.78 (0.75–0.81)	0.80 (0.77–0.83)	0.22
	0.2	0.01	0.75 (0.69–0.81)	0.35 (0.30–0.40)	0.94 (0.91–0.97)	0.14
		0.05	0.84 (0.82–0.87)	0.72 (0.69–0.76)	0.88 (0.86–0.90)	0.26
		0.2	0.78 (0.75–0.81)	0.73 (0.70–0.77)	0.78 (0.75–0.81)	0.20

*Polygenicity* proportion of causal variants of all simulated variants, *AUC* area under the ROC curve, *PPV* positive predictive value, *NPV* negative predictive value, *Coverage* proportion of samples predicted as case or control, *95% CI* 95% confidence interval.

### Applications of MCCP to complex diseases

We used MCCP to evaluate the capacity of PRS in predicting risk for four common complex diseases (CAD, T2D, IBD, and BRCA; *N* = 276,299) from the UK Biobank^23,24^, and SCZ from a Danish population study (iPSYCH, *N* = 24,072)^25^ using MCCP (Table 2). As for simulated data sets, MCCP predictions were well-calibrated across all studied diseases (Supplementary Fig. 6).

**Table 2:** Description of complex diseases.

Disease	Discovery GWAS	Discovery GWAS sample size (#case/#control)	Prevalence in testing data set(#case/#total)	#Polymorphisms used in PRS construction
CAD	Nikpay et al.^43^	60,801/123,504	13,689/276,299 (5.0%)^a^	9912
T2D	Scott et al.^44^	26,676/132,532	15,006/276,299 (5.4%)^a^	19,054
IBD	Liu et al.^45^	12,882/21,770	2471/276,299 (0.9%)^a^	10,878
BRCA	Michailidou et al.^46^	122,977/105,974	9653/147,317 (6.6%)^a^	28,945
SCZ	PGC^5^	35,642/111,748^b^	5125/24,072 (21.3%)^c^	31,755
T2D (MDC)	Mahajan et al.^42^	74,124/824,006	943/24,298 (3.9%)^d^	126,748

^a^Restricted to European unrelated participants from the UK biobank.

^b^Participants from the Danish sub-cohorts were removed.

^c^European unrelated participants from the Integrative Psychiatric Research (iPSYCH) schizophrenia sample.

^d^European unrelated participants from the Malmö Diet and Cancer (MDC) study at baseline.

*CAD* coronary artery disease, *T2D* type 2 diabetes mellitus, *IBD* inflammatory bowel disease, *BRCA* breast cancer, restricted to women in testing data set, *SCZ* schizophrenia, *T2D (MDC)* T2D data set from the MDC study at the baseline.

**Fig. 3:**
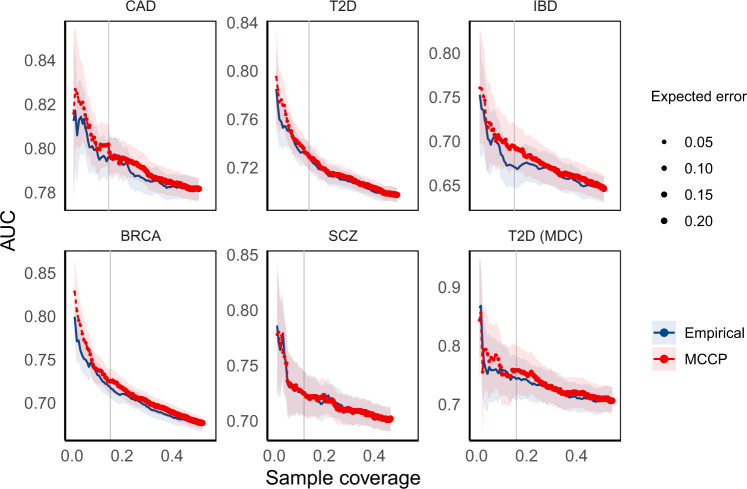
Comparison of the performance of MCCP and the empirical method on complex disease risk prediction using PRS as predictor. For MCCP, sample coverage (*x* axis) indicates the proportion of samples predicted as cases or controls, whereas, for the empirical method, it indicates extreme PRS, e.g., top and bottom x% of PRS. AUCs are computed from multivariate logistic regressions adjusted for age, sex, and PC1–6 on these samples stratified from MCCP and empirical method, respectively. The expected error rates for MCCP are indicated by the size of data points up to 0.20. Vertical lines correspond to an expected error of 0.05 from the MCCP. The solid lines and shades represent the median and 95% confidence intervals of AUCs. *CAD* coronary artery disease, *T2D* type 2 diabetes mellitus, *IBD* inflammatory bowel disease, *BRCA* breast cancer, *SCZ* schizophrenia, *T2D (MDC)* T2D data set from the MDC study at the baseline. Source data are provided as a Source Data file.

A commonly used approach for making decisions in trait prediction is contrasting the top x% with the bottom x% of PRS (termed empirical method). We compared the performance of MCCP to that of the empirical method in disease risk prediction with a fixed coverage, i.e., the number of predictable subjects in a testing sample. As the empirical method does not formally use the concept of coverage, we defined it as two times the extreme x%. Using PRS as the only predictor, we found that MCCP performed marginally better than the empirical method in all studied diseases at error rates ranging from 0 to 0.2 (Fig. 3). A key advantage of using MCCP is that it can estimate confidence bounds for individual prediction. Based on these individualized predictions, MCCP can help clinicians make a decision at an error rate *α*.

**Fig. 4:**
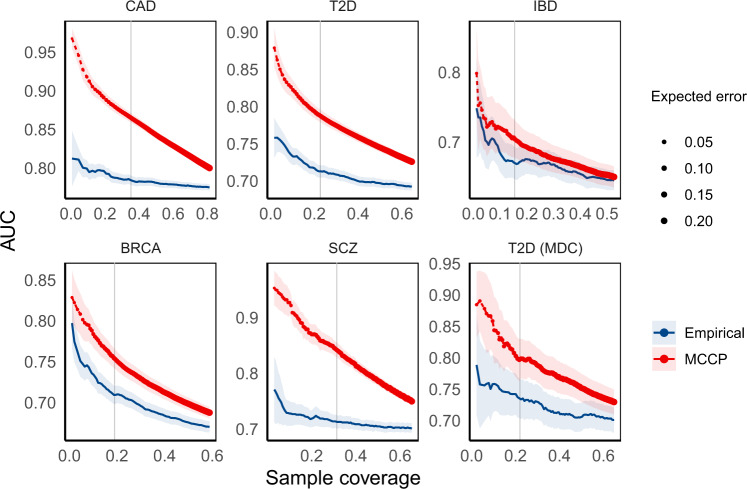
Comparison of the performance of MCCP and the empirical method on complex disease risk prediction using PRS and additional information (age, sex, and PC1–6) in MCCP. For MCCP, sample coverage (*x* axis) indicates the proportion of samples predicted as cases or controls, whereas, for the empirical method, it indicates extreme PRS, e.g., top and bottom x% of PRS. AUCs are computed from multivariate logistic regressions adjusted for age, sex, and PC1–6 on these samples stratified from MCCP and empirical method, respectively. The expected error rates for MCCP are indicated by the size of data points up to 0.20. Vertical lines correspond to an expected error of 0.05 from the MCCP. The solid lines and shades represent the median and 95% confidence intervals of AUCs. *CAD* coronary artery disease, *T2D* type 2 diabetes mellitus, *IBD* inflammatory bowel disease, *BRCA* breast cancer, *SCZ* schizophrenia, *T2D (MDC)* T2D data set from the MDC study at the baseline. Source data are provided as a Source Data file.

We, next, evaluated the performance of MCCP when age, sex, and genetic ancestry information (computed by genetic principal components PC1–6, see Methods) were included in model building, calibration, and prediction steps. We found that MCCP outperformed the empirical method in studied data sets, which had varying case-control ratios from 1:4 to 1:111 (Fig. 4 and Supplementary Fig. 7). Such excellent performances were especially apparent for CAD, T2D, and SCZ. As expected, AUC, PPV, and NPV from MCCP decreased with increased coverage and with a decreasing preset confidence level. At an error rate of 0.05, MCCP can predict 35.2% of subjects for CAD, 22.7% for T2D, 15.5% for IBD, 19.0% for BRCA from the UK Biobank, and 31.4% of subjects of iPSYCH SCZ data set either as cases or controls. The prediction accuracies measured by AUCs were 0.865 (CAD, 95% CI 0.859–0.871), 0.788 (T2D, 95% CI 0. 779–0.796), 0.705 (IBD, 95% CI 0.678–0.732), 0.754 (BRCA, 95% CI 0.742–0.766) and 0.842 (SCZ, 95% CI 0.828–0.856), respectively (Supplementary Table 1). In contrast to MCCP, the empirical method at a coverage of 10% (i.e., top 5% vs bottom 5% of PRS), gave 0.01–0.11 less in AUCs. Importantly, the empirical method cannot report confidence levels for risk prediction.

### Validation of MCCP prediction by a follow-up study

Because complex diseases have different ages of onsets between individuals, some healthy controls at the time of prediction may develop the disease later in life, we further examined the performance of MCCP in prediction using the MDC T2D follow-up data set^26^. At baseline, median ages for men, women, and both sexes were 59.1 years (interquartile range [IQR]: 53.1–64.6), 57.4 (IQR: 50.1–63.7), and 57.8 (IQR: 51.3–64.2). T2D status of subjects in this data set was followed for 20 years and assessed at five time points, i.e., baseline, year 5, 10, 15, and 20. We estimated the prediction probability values at baseline using MCCP and the empirical method, and then, evaluated the performance of these predictions at each of the four follow-ups, using the corresponding T2D status or censored. In line with the results for the cross-sectional T2D data set from UK Biobank, MCCP slightly outperformed the empirical method when using PRS as the only predictor (Fig. 3 and Supplementary Fig. 8) but outperformed the empirical method strikingly at baseline using MDC data set (Fig. 4 and Supplementary Fig. 7) when age, sex, and genetic PC were considered in MCCP. When these covariates were considered in MCCP, the improved performances of MCCP also hold in the follow-up years 5, 10, 15, and 20 (Supplementary Fig. 9). Not surprisingly, MCCP also showed improved PPVs and decreasing NPVs along with follow-ups (Supplementary Figs. 10 and 11). Although a decreasing AUC with follow-up time was observed for MCCP, it still performed better than the empirical method at all studied time points (Supplementary Figs. 9a, 10a, and 11a). We also trained and applied MCCP and the empirical method at each time point of follow-ups. Results from these models consistently suggested a better performance of MCCP than that from the empirical method (Supplementary Figs. 9b, 10b, and 11b).

## Discussion

In the present study, we introduce the machine-learning method MCCP as a calibration tool within the current polygenic risk prediction methodology. As a proof of concept, we show that MCCP is capable of estimating confidence levels for an individual’s predicted risk. The estimates obtained with MCCP enjoy the validity property, i.e., in long run, the prediction errors are guaranteed to fall below a preset error rate^27^. The estimated probability values for hypothetically assigning an individual as a case or control will have more direct utility in clinics than group-wise estimates, which arbitrarily define the top 10%, 5%, or 1% of samples as the high-risk group, and, similarly the bottom x% as a healthy group. In addition, with a preset error rate, e.g., *α* = 0.05, MCCP can systematically estimate the proportion of samples that can be predicted with an error rate below α. Such functionality is extremely valuable given that the performance of PRS depends on genetic architectures of complex diseases, which are typically unknown. For example, with a desired prediction error rate of 0.05, for one disease, 10% of samples can be reliably predicted by MCCP; but for others, only 5% may be predictable. In its current form, PRS is designed to predict risk at the population or group level^30^, whereas ideally, one would want to know the individual’s susceptibility to disease. Thus, MCCP could aid clinicians in diagnosis by telling the prediction confidence for each individual.

Human diseases generally vary in prevalence, which in turn vary in time and across populations. Differences in prevalence between a reference GWAS sample and the tested population need to be considered before choosing the approach to use, such as defining the top x% of PRS in the tested population as the risk group. Our MCCP model can systematically overcome a situation where prevalence between GWAS sample and the tested population is different. Using a calibration set, MCCP estimates personalized confidences of prediction being case and control status, respectively. As such, MCCP can provide the users confidences on how much of their sample can be accurately predicted, no matter case or controls. Knowing the probability of being healthy (low risk of developing a disease) is equally important for the sake of public health management^31,32^. In the present analysis, we observed a well-calibrated confidence level for predictions of common disorders in UK biobank data sets, SCZ in a Danish data set, and T2D in a Swedish data set. We also explored the proportion of samples that can be predicted within a given error rate. Thus, using confidence of prediction at various expected error rates to make decisions seems feasible.

Another advantage of MCCP is that it is indifferent to the bias of an imbalanced case-control study design^28^. As the percentage of years lived with disability (% YLDs) for most complex diseases or conditions are <10%^33^, data imbalance is common in population-based studies. Such study design typically results in a majority class and a minority class in the sample. In extreme cases, even a simple predictor that treats all samples as the majority class can have good accuracy but fail to predict in the minority class. MCCP handles imbalanced data without the need to consider explicit balancing measures, such as over- or under-sampling. Our simulations and real data applications for complex diseases showed that MCCP performs remarkedly well and its validity was preserved even when the data were severely imbalanced, e.g., with prevalence ranging from 0.01 to 0.2, which reflected an imbalance level ranging from 1:99 to 1:4.

We demonstrated that predictions made by MCCP reflected the lifetime probability of being case or control using the MDC follow-up studies. As individuals typically show varying ages of onset for a specific disease, some healthy controls at the time of recruitment in a study may later develop such disease. Assigning probability values to an individual of being case or control at baseline using MCCP allows us to identify high-risk individuals. Meanwhile, such functionality automatically makes the MCCP prediction testable in the future. Our application to the 20 years follow-up data set from MDC showed that the prediction made by MCCP became more accurate as follow-up continues, confirming the applicability of MCCP. In addition, directly applying MCCP at each follow-up time point further improves the prediction performance both in prediction error rate and sample coverage. Surprisingly, these crucial functions have not been formally developed by the empirical method.

We implemented MCCP onto PRS as an extension to the polygenic risk prediction paradigm such that advances in constructing PRS will also improve the performance of MCCP in personalized risk prediction. It has been shown that directly modeling linkage disequilibrium structure among SNPs and combining PRS constructed from different diseases or traits may improve the predictability of PRS^34–36^. Thus, we hypothesize that incorporating these advanced PRS models into our implementation will further improve the performance of MCCP. Moreover, the major improvement achieved by MCCP is the calibration step. Thus, the performance of MCCP could also be improved by constructing a disease risk score combining both genetic and non-genetic factors, such as lifestyle, socioeconomic status, and environmental exposures. We used logistic regression as our underlying model to construct a nonconformal measure (NCM). Other machine-learning models, such as k-nearest neighbors, random forest, support vector machine, or deep neural networks, can also be used to improve the overall power of MCCP in risk prediction.

In this work, we evaluated the performance of MCCP on samples of European ancestry. However, the application of our method in trans-ethnicity (e.g., training on European population and testing on Africa or Asian population) should be an interesting next step.

In conclusion, we described an approach for personalized genetic risk assessment of complex diseases. By estimating personalized confidences of risk prediction, it can help clinical professionals to assess the value of genetic data in disease risk prediction.

## Methods

### Samples for UK Biobank, iPSYCH, and MDC

The UK Biobank project is a prospective cohort study, composing of ~500,000 individuals from the United Kingdom aged between 40 and 69 at recruitment^23,24^. Participants were genotyped using the Affymetrix UK BiLEVE Axiom array and the Affymetrix UK Biobank Axiom array, respectively. Quality control, ancestral origins, and cryptic relatedness were described elsewhere^24^. Phenotypes of CAD, T2D, IBD, and BRCA were retrieved using ICD9/10 codes, operation and procedure codes from hospital inpatient records (UK Biobank fields 41270, 41271, and 41272), as well as self-reported medical conditions and procedures (UK Biobank fields 20001, 20002, 20004). After standard quality, 276,299 unrelated participants with European ancestry remained for further analysis. The UK Biobank received approval from the National Information Governance Board for Health and Social Care and the National Health Service North West Center for Research Ethics Committee (Ref: 11/NW/0382). This research has been conducted using the UK Biobank Resource under application number 32048.

A detailed description of the iPSYCH cohort has been reported elsewhere^25^. In brief, the iPSYCH is a representative sample of the entire Danish population born between 1981 and 2005, including 1,472,762 subjects. Initial genotyping was performed at the Broad Institute with amplified DNA extracted from dried blood spots and assayed on the Infinium PsychChip v1.0 array. SNPs were phased into haplotypes using SHAPEIT3 and imputed using Impute2 with European reference haplotypes from the 1000 genomes project phase 3^37^. Individuals were censored to ensure no pair has closer than third-degree kinship. In total, 5125 cases of SCZ (ICD10 code F20) and 18,947 controls with imputed genotypes were used for the present study. iPSYCH was approved by the Danish Scientific Ethics Committee, the Danish Health Data Authority, the Danish Data Protection Agency, Statistics Denmark, and the Danish Neonatal Screening Biobank Steering Committee. In accordance with Danish legislation, this study has waived the need for informed consent in biomedical research based on existing biobanks by the Danish Scientific Ethics Committee.

The MDC study is a population-based prospective cohort study in southern Sweden. Details of the cohort and the recruitment are described elsewhere^26^. All participants were followed until incident diabetes, emigration from Sweden, death, or the end of follow-up (31 December 2016), whichever came first. Information on new diabetes cases was retrieved from both local and national registers^38^. To include all-possible T2D participants, all cases that were specified as type 1, LADA, secondary diabetes or others, were discarded from further analysis. Subjects without screening date at baseline or diabetic patients with the first event of diabetes before the age of 40 years were also excluded. The application of these criteria resulted in 5647 cases of T2D and 24,011 non-diabetic controls. Genotyping was performed on the Illumina GSA v1 genotyping array with amplified DNA extracted from the whole-blood sample. All procedures followed the standard protocol. The genotyped SNPs were excluded for the probe to genome mismatch, incorrect assignment of allelic variants in the array design, MAF < 0.01, failed Hardy–Weinberg Equilibrium test at *p* < 1 × 10^−15^, call rate <99%, or failed genotype calling. Samples were excluded if they showed evidence of gender mismatch or had an overall sample call rate <90%. Kinship was estimated using KING v2.2.4^39^ and individuals were censored to ensure no pair had closer than third-degree kinship. Imputation was performed using the Michigan Imputation Server with the reference panel of Haplotype Reference Consortium^40^ (HRC r1.1).

We restricted our analysis to MDC European populations and censored non-diabetic participants if they lost follow-up, emigrated, or died within follow-up years 5, 10, 15, and 20. This resulted in 943, 1611, 2559, 3695, and 4277 cases of T2D at each time point, and the total numbers of participants per time point are 24,298, 23,211, 21,936, 20,247, and 16,146, respectively. Here, we implemented MCCP in two ways: (1) model building at baseline, and prediction at baseline and re-examined at each of the four follow-ups with respective T2D status. (2) model building and prediction at five time points, respectively.

MDC was approved by the Ethics Committee of Lund University (LU 51–90) and all patients provided written informed consent. The use of data in this study was approved by the MDC steering Committee.

### Simulation of different genetic architectures

To examine the applicability of MCCP in various situations of GWAS design, we simulated case-control phenotypes based on real genotypes on chromosome 2 of 10,000 individuals from the iPSYCH project. The synthetic phenotypes were generated by combinations of varying polygenicity of 0.001, 0.01, 0.1, and 0.2, the heritability of 0.3, 0.5, and 0.8, and prevalence of 0.01, 0.05, 0.1, and 0.2 using the GCTA software^41^. The prevalence rate was chosen based on the reported prevalence of the considered diseases^33^. GWAS was performed using sample proportions of 0.2, 0.5, and 0.7 as discovery data sets. Consequently, PRS was obtained by a thresholding (*p* < 0.05) and pruning (LD *r*^2^ < 0.1) approach. Logistic regressions within the MCCP setting were performed in the calibration and testing samples.

### PRS construction

PRS was computed as the weighted sum of effect for the pruned SNPs (LD *r*^2^ < 0.1) with MAF ≥ 0.01 in the target individuals. Indels and variants in the extended MHC region (build hg19, chromosome 6: 25–34 Mb) were removed. Effect sizes were taken from the reference GWAS or from the discovery set in simulation studies. In this study, summary statistics were from Psychiatric Genomics Consortium phase 2 without the Danish sub-cohorts^5^ and DIAGRAM^42^ (2018) for iPSYCH SCZ and MDC T2D data sets (MAF ≥ 0.05), respectively. For CAD, T2D, IBD, and BRCA from UK Biobank, we used summary statistics from respective GWAS studies^43–46^ where UK Biobank samples were not included, same to a recent study^9^. PLINK^47^ was used to construct PRS with the following parameters: *p* value < 0.05 and *r*^2^ threshold of 0.1 within a window size of 100 kb and step size of 50 bp. And the LD structure from the 1000 Genomes Project phase 3 European subpopulation was used for LD pruning.

### Logistic regression

As proof of principle, we built models based on simple lR to make a prediction. In simulation studies, LR was performed by simply using PRS alone as an independent variable. In the real-world clinical studies, LR was performed using PRS as an independent variable and age, sex, genotyping batches, and the first six PCs of population structures as covariates.

### CP and MCCP

CP estimates the confidence of predicting the class *y*_*i*_ to a new object *x*_*i*_ given a training set of *z*_1_(*x*_1_, *y*_1_), *z*_2_(*x*_2_, *y*_2_), … *z*_1_(*x*_n_, *y*_n_), where *x*_i_ is generally a vector and *y*_i_ is two-class labels indicating the class to which the *x*_i_ belongs. A measurable function (equation [2]) quantifies how unusual (nonconformal) the *x*_i_ is in comparison with the training set,

2 $${{{{{\rm{NCM}}}}}}_{y}=-y* d({x}_{i})$$

where NCM is nonconformity measure, *y* is the all-possible nonzero classes, e.g., (1, −1) and *d*(*x*_i_) is the decision value obtained from the decision function of the fitted model, e.g., LR in the present study. As in the definition of the inductive conformal prediction of CP, the training set is further split into a proper training set (*z*_1_,…,*z*_*l*_) and a calibration set (*z*_*l*_,…,*z*_n_) where *i* is less than *n*. NCM is calculated for both calibration and test sets based on the model trained on the proper training set. We assume that both training and calibration sets are independent and identically distributed. Using the only calibration set alone, the probability value of an individual *i* to be the class *y* is calculated as follows

3 $${p}_{y}^{i}=\frac{|\{j=1,\,\ldots ,\,N\_{{{{{\rm{cal}}}}}}:\,{{{{{\rm{NCM}}}}}}_{y,j}\ge {{{{{\rm{NCM}}}}}}_{y,i}\}|}{(N\_{{{{{\rm{cal}}}}}}+1)}$$

Note that all samples in the calibration set are included to compute the probability values for all-possible classes. This may be problematic when the data are imbalanced. To address this issue in the classification model, the MCPP was introduced^28^. As shown in Eq. (1), MCCP restricts NCM comparisons with the calibration set within sample class (e.g., case or control alone in binary classifications). By choosing an expected error *α* ∈ [0, 1], for every test sample, a predicting region outputs the following:

4 $${\Gamma }^{\alpha }=\{y\in Y:{p}_{y}\, > \, \alpha \}$$

where *Y* is the set of possible classes, *p*_*y*_ is the probability value when an individual is in a class. In the binary classification problem, we set *p*_1_ as the probability value when an individual is in one class, e.g., case, and *p*_0_ as another class, e.g., control. Unlike other classification frameworks, where a prediction is always provided and is a unique class, the prediction region Γ^*α*^ is a set and it can be empty or contain one or two classes. Confidence, credibility, and prediction of MCCP are defined as follows:

confidence: ${{{{{\rm{sup}}}}}}\{1-\alpha :\,{\Gamma }^{\alpha }\le 1\}$, that is the greatest 1–*α* for which Γ^*α*^ is a unique class, e.g., case or control. In the problem of binary classifications, it is also equal to 1− min(*p*_0_, *p*_1_).

credibility: $\{\alpha :\,|{\Gamma }^{\alpha }|=\,0\,\}$, i.e., the smallest *α* for which *Γ*^*α*^ is empty. It is also equivalent to max(*p*_0_, *p*_1_) in the binary classifications.

prediction: Γ^*α*^ when 1−*α* is equal to the confidence, i.e., *α* = min(*p*_0_, *p*_1_) in binary classifications.

As an example of interpretation, given an output *p*_0_ of 0.01 and *p*_1_ of 0.8 for a test individual, the individual will be predicted to be a case with a credibility of 0.8 and confidence of 0.99.

### Calibration assessment

A reliability curve (observed error versus expected error) was used to assess calibrations by MCCP and an LR model. First, we divided data into five folds. MCCP and LR were built on four folds and used to make predictions on the remaining fold. This procedure was repeated five times to make sure all samples were covered. The observed error was measured as the proportion of incorrect predictions against true cases or controls status at an expected error rate using the estimated MCCP probability values and probabilities from the LR, respectively. At a given error rate (so-called expected error rate) ī, the observed error for LR and MCCP was computed by Eqs. (5) and (6), respectively.

5 $${{{{{\rm{Observed}}}}}}_{{{{{\rm{err}}}}}}{{{{{\rm{(LR)}}}}}}=\sum \{{p}^{i} \, > \, (1.0-\alpha ):y=0\Vert {p}^{i}\, < \, \alpha :y=1\}\,$$

6 $${{{{{\rm{Observed}}}}}}_{{{{{\rm{err}}}}}}{{{{{\rm{(MCCP)}}}}}}=\sum \{{p}_{y}^{i}\le \,\alpha :y\}$$

### Evaluation metrics

Performances of MCCP are measured by validity and coverage (also termed efficiency). A valid prediction means that the frequency of errors (i.e., the fraction of true values outside the prediction region) is no more than *α* at a chosen error rate *α*. The validity can be calculated for all class objects as well as for objects of one specific class. Coverage is defined as the percentage of unique class Γ^*α*^, which is also the proportion of samples predicted as case or control, as shown in this study. Varying error rate α from 0 to 1, observed error, coverage, AUC, PPV, and NPV are computed using LR adjusted for age, genetic sex, batches of genotyping arrays, and the first six PCs of population structures. Confidence intervals of AUC, PPV, and NPV were calculated using the pROC package within R.

### Reporting summary

Further information on research design is available in the Nature Research Reporting Summary linked to this article.

## Supplementary information

Supplementary Information

Reporting Summary

## Data Availability

All GWAS summary statistics used in this study are publicly available in the following repositories: Coronary ARtery DIsease Genome wide Replication and Meta-analysis plus The Coronary Artery Disease Genetics consortium (coronary artery disease), http://www.cardiogramplusc4d.org/data-downloads/; DIAbetes Genetics Replication And Meta-analysis consortium (type 2 diabetes mellitus), https://diagram-consortium.org/downloads.html; International Inflammatory Bowel Disease Genetics Consortium (inflammatory bowel disease), https://www.ibdgenetics.org/downloads.html; Breast Cancer Association Consortium (breast cancer), http://bcac.ccge.medschl.cam.ac.uk/bcacdata/oncoarray/oncoarray-and-combined-summary-result/; Psychiatric Genomics Consortium (schizophrenia), https://www.med.unc.edu/pgc/download-results/scz/. Data from 1000 Genomes Project can be accessed at ftp://ftp.1000genomes.ebi.ac.uk/vol1/ftp/. UK Biobank data are available to registered investigators upon approval via http://www.ukbiobank.ac.uk. Data from the MDC study can be applied for access through https://www.malmo-kohorter.lu.se. In accordance with the consent structure of iPSYCH and Danish law, individual-level genotype and phenotype data from the iPSYCH study are not able to be shared publicly. Source data are provided with this paper.

## Code availability

R codes for MCCP are made available for research use at GitHub (https://github.com/sunjiangming/PRS_MCCP) and are archived in Zenodo (10.5281/zenodo.4661464)^48^. Additional software PLINK v1.90b5.2, KING 2.2.4, GCTA v1.25.2 and R packages (glmnet_2.0-16, doParallel_1.0.14, foreach_1.4.4, caret_6.0-82, impute_1.56.0, rms_5.1-2, pROC 1.16.2, Zelig 5.1.7) were used in this study.
