# Supplementary material for: Translating polygenic risk scores for clinical use by estimating the confidence bounds of risk prediction
Source: Nat Commun. 2021 Sep 6;12:5276. doi: 10.1038/s41467-021-25014-7 (PMC8421428; doi:10.1038/s41467-021-25014-7)
Supplement: Supplementary file 1 — Supplementary Information [file 41467_2021_25014_MOESM1_ESM.pdf]

Supplementary Information

Translating polygenic risk scores for clinical use by estimating the confidence bounds of risk prediction

Sun et al.

This file contains:

Supplementary Table 1
Supplementary Figures 1-11
Supplementary Note

Supplemental Table 1. Performances of Mondrian cross-conformal prediction at expected error of 0.05 versus empirical method (top 5% and bottom 5% PRS)

Disease	Method	Coverage (%)	PPV (95% CI)	NPV (95% CI)	AUC (95% CI)
CAD	MCCP	35.2	0.186 (0.183-0.190)	0.992 (0.991-0.992)	0.865 (0.859-0.871)
	Empirical	10	0.118 (0.116-0.120)	0.979 (0.978-0.980)	0.784 (0.778-0.790)
T2D	MCCP	22.7	0.147 (0.144-0.150)	0.984 (0.983-0.985)	0.788 (0.779-0.796)
	Empirical	10	0.112 (0.104-0.120)	0.970 (0.967-0.973)	0.733 (0.725-0.741)
IBD	MCCP	15.5	0.019 (0.017-0.021)	0.996 (0.996-0.997)	0.705 (0.678-0.732)
	Empirical	10	0.017 (0.016-0.019)	0.996 (0.995-0.997)	0.682 (0.659-0.705)
BRCA	MCCP	19.0	0.145 (0.140-0.149)	0.976 (0.974-0.978)	0.754 (0.742-0.766)
	Empirical	10	0.138 (0.134-0.142)	0.972 (0.969-0.975)	0.734 (0.720-0.749)
SCZ	MCCP	31.4	0.345 (0.327-0.362	0.961 (0.957-0.964)	0.842 (0.828-0.856)
	Empirical	10	0.341 (0.330-0.351)	0.870 (0.862-0.878)	0.728 (0.706-0.750)

Analysis on breast cancer is restricted to female participants. CAD: Coronary artery disease; T2D: Type 2 diabetes mellitus; IBD: Inflammatory bowel disease; BRCA: Breast cancer; SCZ: Schizophrenia; PPV: positive predictive value, NPV: negative predictive value, AUC: Area under the curve; 95% CI: 95% confidence interval.

Supplemental Figure 1. Calibration plots comparing observed error and expected errors from MCCP and the logistic regression model using PRS as predictor. The observed error is measured as the proportion of incorrect predictions against true cases or controls status at an expected error rate using the estimated MCCP probability values and probabilities from the logistic regression, respectively. Logistic regression is fitted on the combined proper training and calibration sets (same as that for MCCP) and predicted on the test set. Optimal calibration is shown as dashed line which is largely overlapped with MCCP curves. Proportion of GWAS discovery set is 0.5. Source data are provided as a Source Data file.

(a) heritability: 0.3

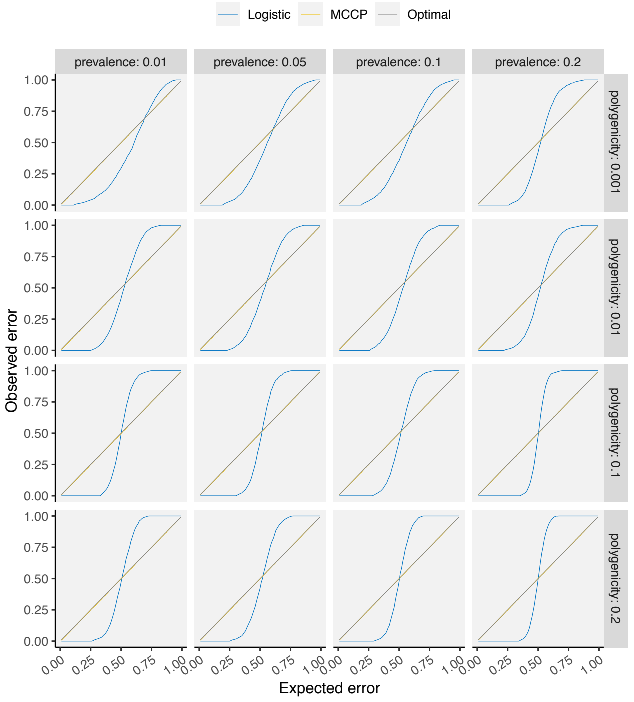

(b) heritability: 0.5

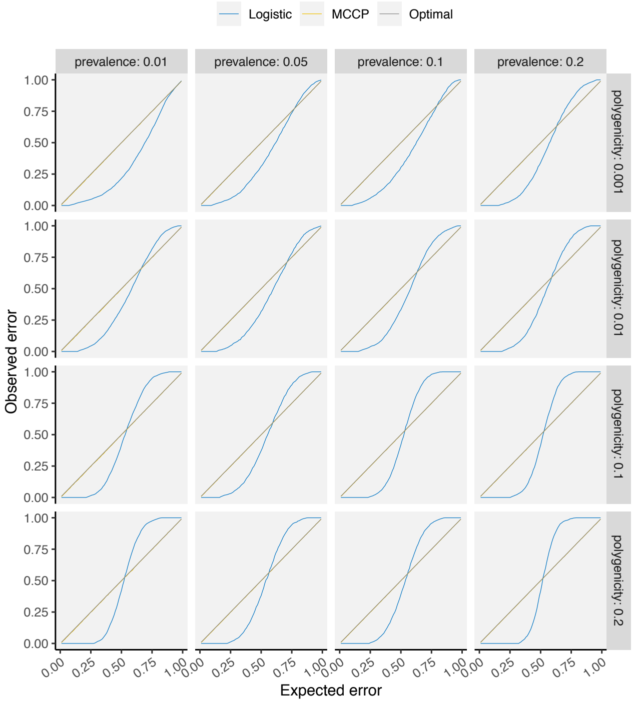

(c) heritability: 0.8

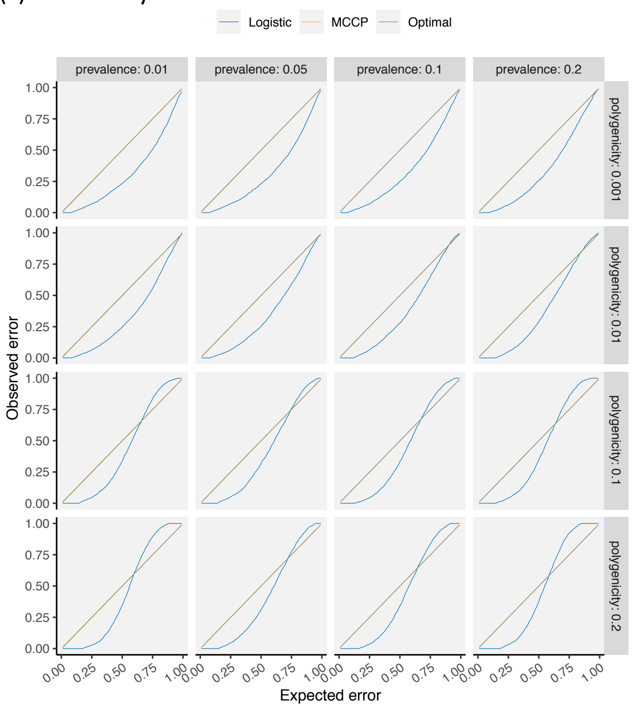

Supplemental Figure 2. The observed errors from Mondrian cross-conformal prediction versus expected errors using PRS as predictor for all combinations of heritability (h^2), prevalence, polygenicity and proportion of discovery set. Source data are provided as a Source Data file.

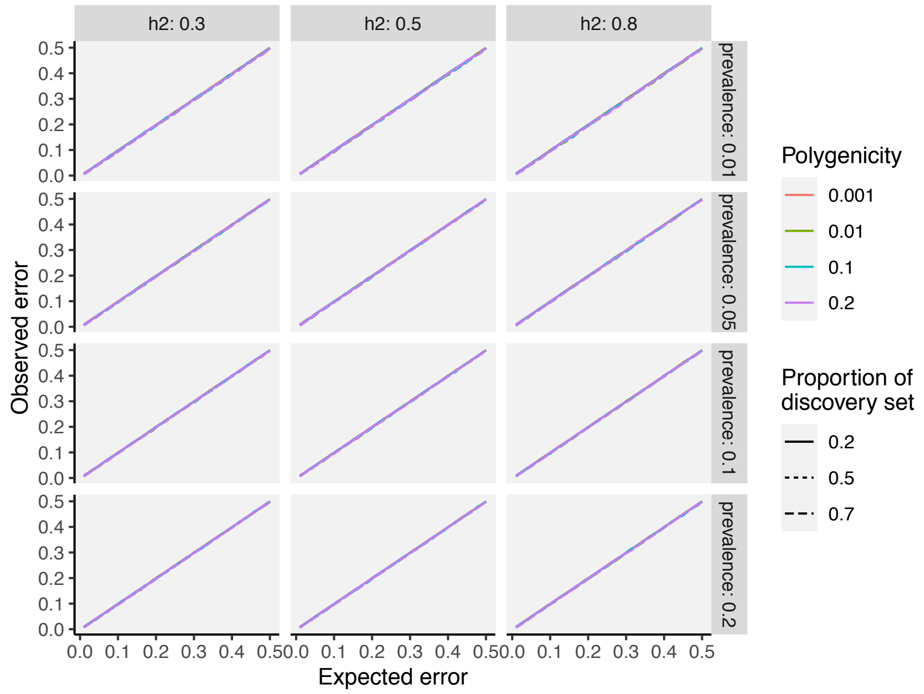

Supplemental Figure 3. The observed errors from Mondrian cross-conformal prediction versus expected errors using PRS as predictor for all combinations of heritability (h^2), prevalence, polygenicity and proportion of discovery set restricted to case set. Source data are provided as a Source Data file.

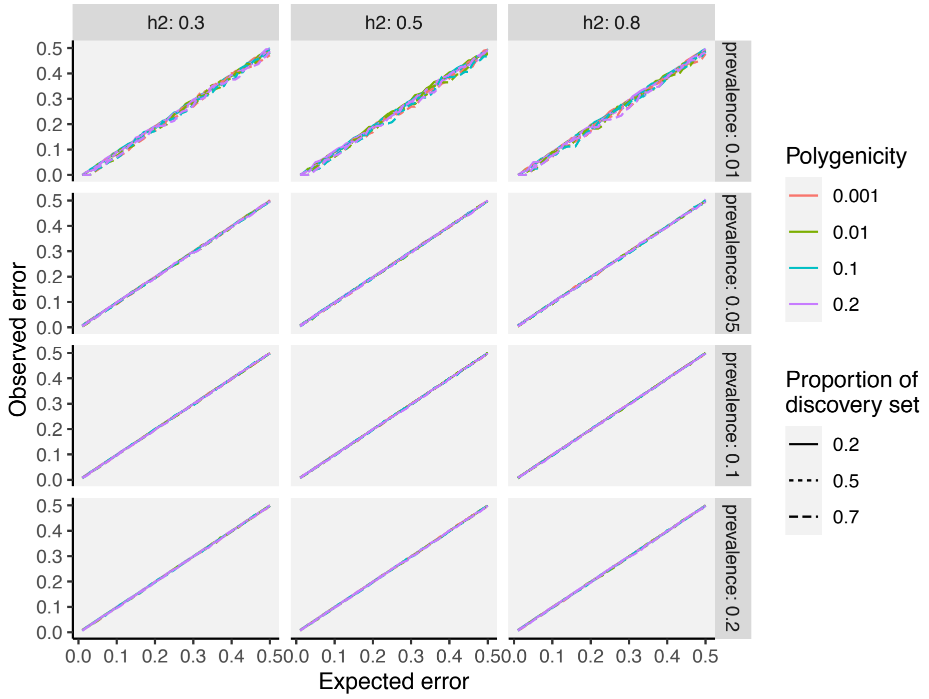

Supplemental Figure 4. The observed errors from Mondrian cross-conformal prediction versus expected errors using PRS as predictor for all combinations of heritability (h^2), prevalence, polygenicity and proportion of discovery set restricted to control set. Source data are provided as a Source Data file.

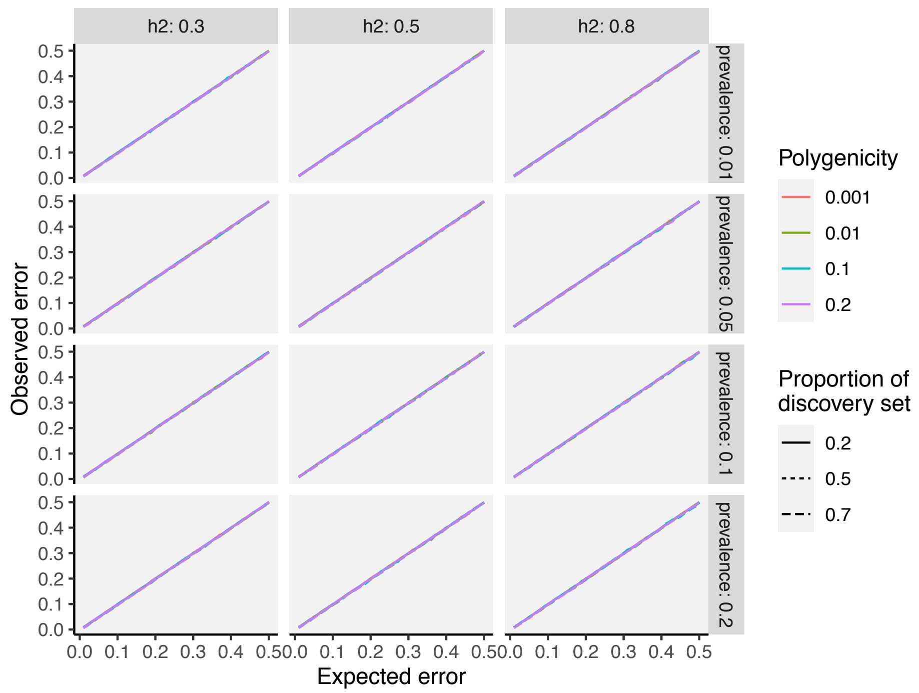

Supplemental Figure 5. Sample coverage obtained from Mondrian cross-conformal prediction using PRS as predictor for all combinations of heritability (h^2), prevalence, polygenicity and proportion of discovery set. Source data are provided as a Source Data file.

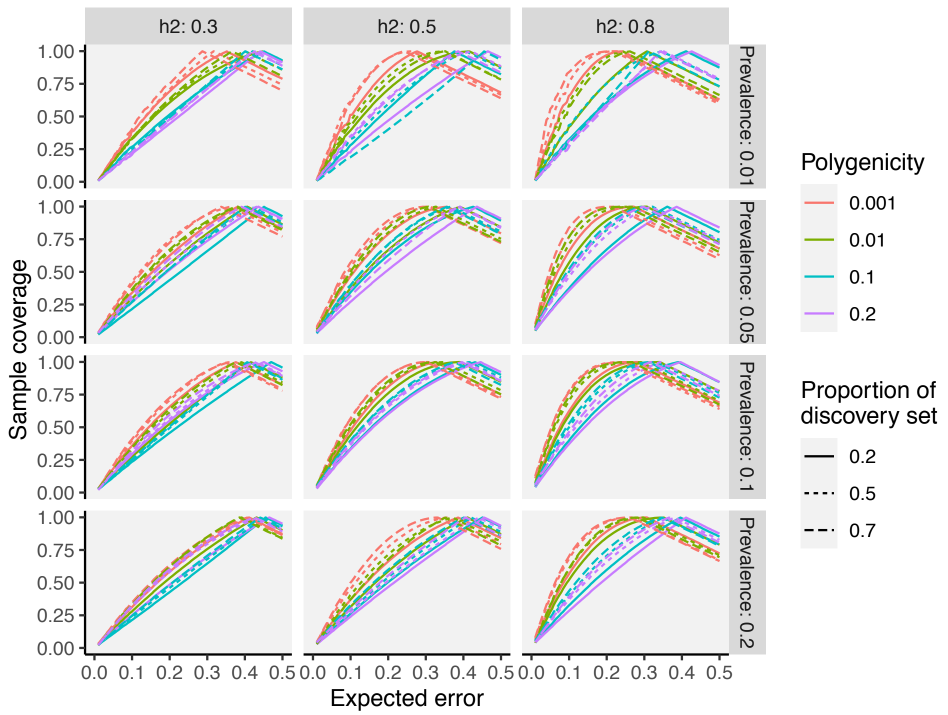

Supplemental Figure 6. Calibration plots comparing observed error and expected errors from MCCP and the logistic regression model on complex diseases. PRS, age, sex and PC1-6 are included in both models. CAD: Coronary artery disease; T2D: Type 2 diabetes mellitus; IBD: Inflammatory bowel disease; BRCA: Breast cancer; SCZ: Schizophrenia; T2D (MDC): T2D dataset from the MDC study at the baseline. Source data are provided as a Source Data file.

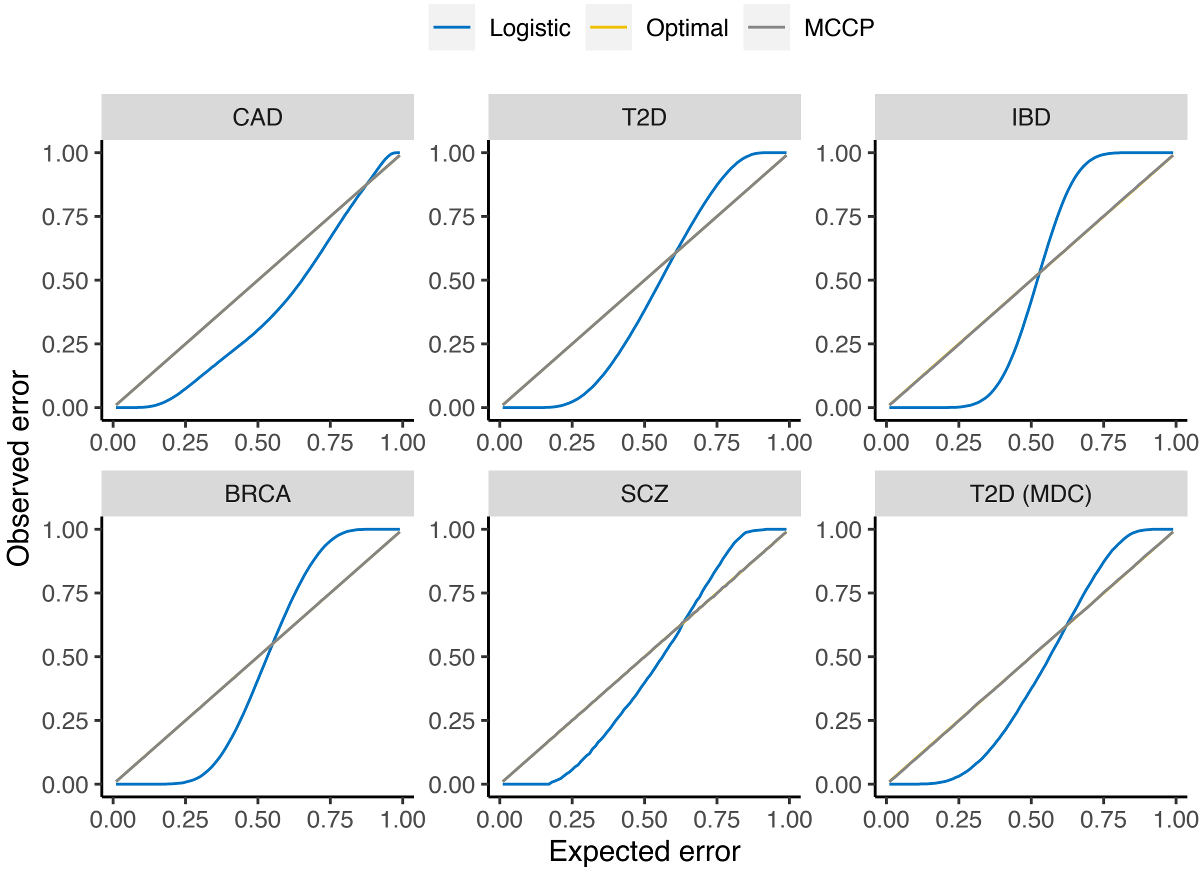

Supplemental Figure 7. Comparison of the performance of MCCP and the empirical method on complex disease risk prediction using PRS and additional information (age, sex, and genetic principal components PC1-6) in MCCP. (a) Positive predictive value (PPV) and (b) Negative predictive value (NPV). For MCCP, sample coverage (x axis) indicates the proportion of samples predicted as cases or controls, whereas, for the empirical method, it indicates extreme PRS, e.g., top and bottom x% of PRS. The expected error rates for MCCP are indicated by the size of data points up to 0.20. Vertical lines correspond to an expected error of 0.05 from the MCCP. The solid lines and shades represent the median and 95% confidence intervals for PPV and NPV, respectively. CAD: Coronary artery disease; T2D: Type 2 diabetes mellitus; IBD: Inflammatory bowel disease; BRCA: Breast cancer; SCZ: Schizophrenia; T2D (MDC): T2D dataset from the MDC study at the baseline. Source data are provided as a Source Data file.

(a) PPV

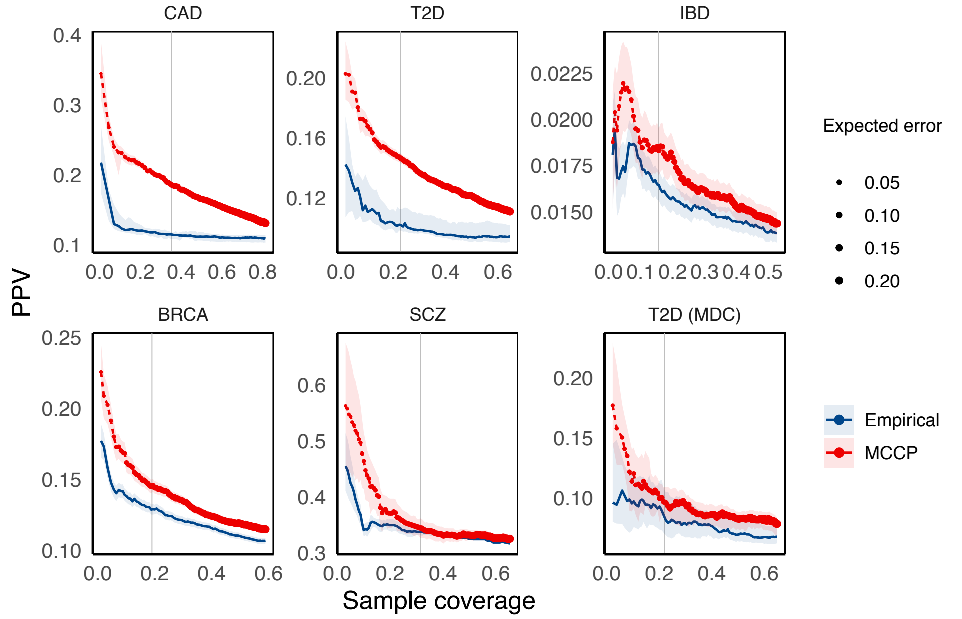

(b) NPV

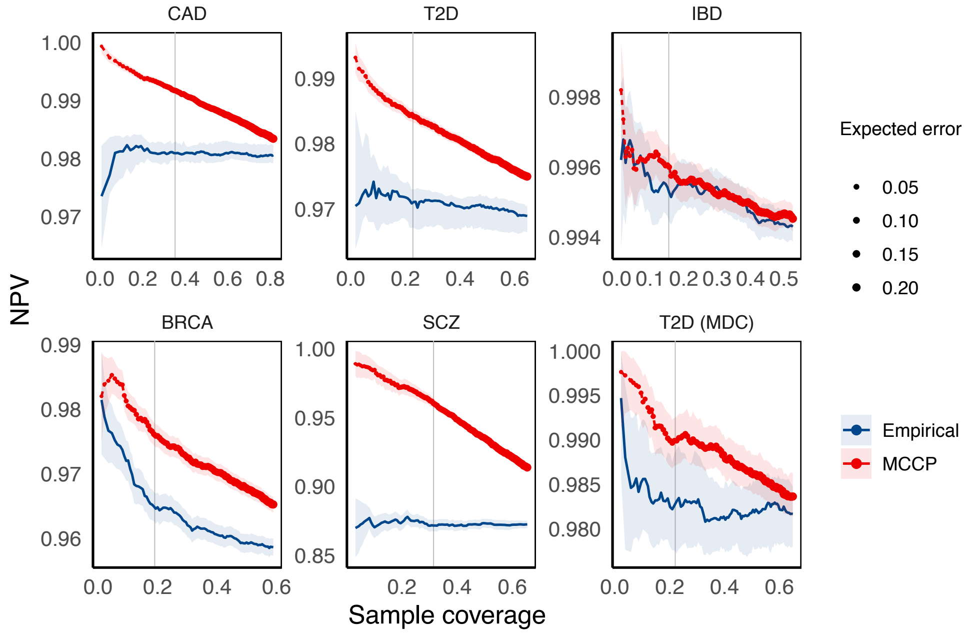

Supplemental Figure 8. Comparison of the performance of MCCP on T2D status in follow-ups (at year 5, 10, 15 and 20) with the empirical method using PRS as predictor. a) AUCs in follow-up year 5, 10, 15 and 20, using models of MCCP and the empirical method, built at baseline. b) AUCs for MCCP and the empirical method built at each time point. MCCP and empirical method are implemented at follow-up year 5, 10, 15 and 20, respectively. For MCCP, sample coverage (x axis) indicates the proportion of samples predicted as cases or controls, whereas, for the empirical method, it indicates extreme PRS, e.g., top and bottom x% of PRS. AUCs are computed from multivariate logistic regressions adjusted for age, sex, and PC1-6 on these sample stratified from MCCP and empirical method, respectively. The expected error rates for MCCP are indicated by the size of data points up to 0.20. Vertical lines correspond to an expected error of 0.05 from the MCCP. The solid lines and shades represent the median and 95% confidence intervals of AUCs. N is total number of participants and N case is number of T2D patients. Source data are provided as a Source Data file.

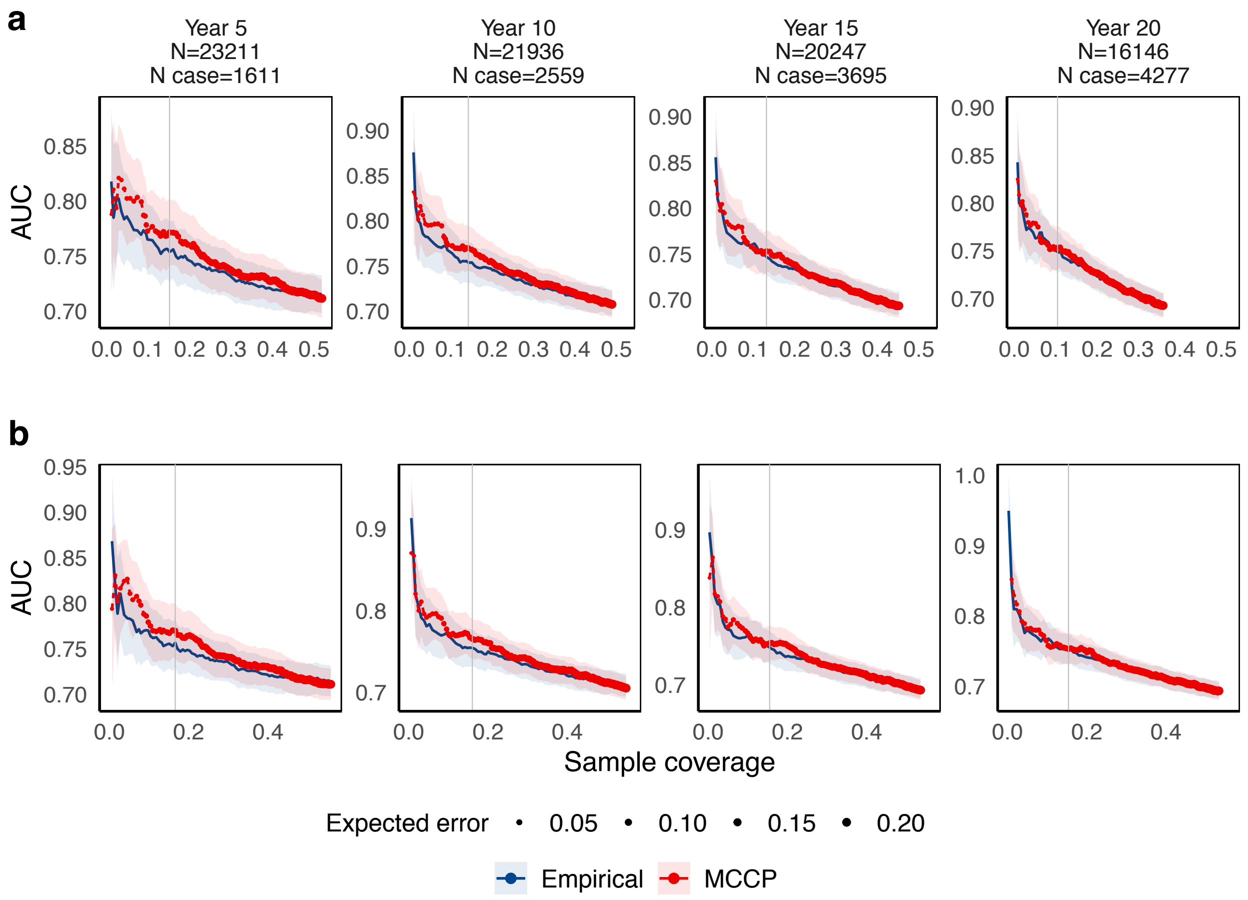

Supplemental Figure 9. Comparison of the performance of MCCP on T2D status in follow-ups (at year 5, 10, 15 and 20) with the empirical method using PRS and additional information (age, sex and PC1-6) in MCCP. a) AUCs in follow-up year 5, 10, 15 and 20, using models of MCCP and the empirical method, built at baseline. b) AUCs for MCCP and the empirical method built at each time point. MCCP and empirical method are implemented at follow-up year 5, 10, 15 and 20, respectively. For MCCP, sample coverage (x axis) indicates the proportion of samples predicted as cases or controls, whereas, for the empirical method, it indicates extreme PRS, e.g., top and bottom x% of PRS. AUCs are computed from multivariate logistic regressions adjusted for age, sex, and PC1-6 on these sample stratified from MCCP and empirical method, respectively. The expected error rates for MCCP are indicated by the size of data points up to 0.20. Vertical lines correspond to an expected error of 0.05 from the MCCP. The solid lines and shades represent the median and 95% confidence intervals of AUCs. N is total number of participants and N case is number of T2D patients. Source data are provided as a Source Data file.

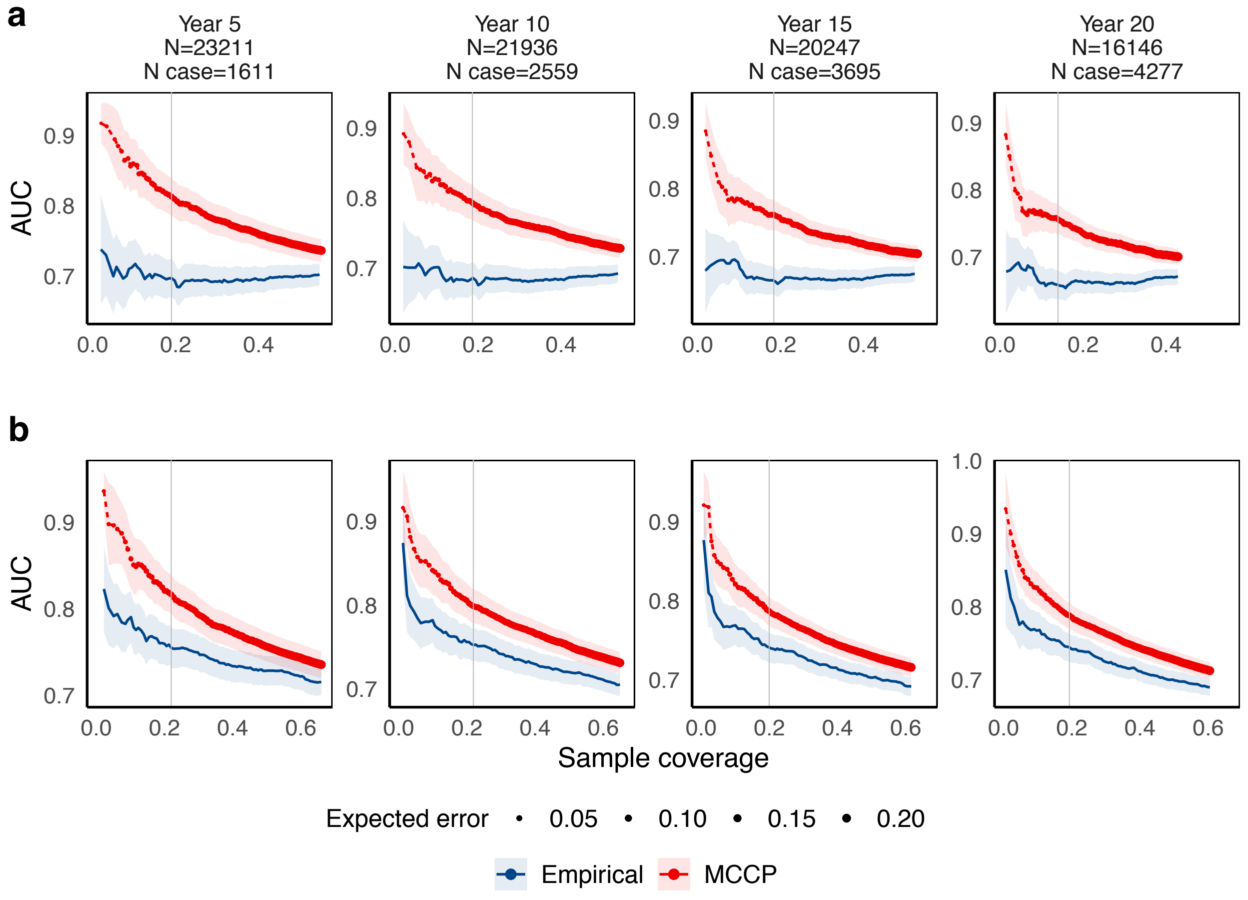

Supplemental Figure 10. Comparison of the performance of MCCP on T2D status in follow-ups (at year 5, 10, 15 and 20) with the empirical method using PRS and additional information (age, sex and PC1-6) in MCCP. a) PPVs in follow-up year 5, 10, 15 and 20, using models of MCCP and the empirical method, built at baseline. b) PPVs for MCCP and empirical method built at each time point. MCCP and empirical method are implemented at follow-up year 5, 10, 15 and 20, respectively. For MCCP, sample coverage (x axis) indicates the proportion of samples predicted as cases or controls, whereas, for the empirical method, it indicates extreme PRS, e.g., top and bottom x% of PRS. The expected error rates for MCCP are indicated by the size of data points up to 0.20. Vertical lines correspond to an expected error of 0.05 from the MCCP. The solid lines and shades represent the median and 95% confidence intervals of PPVs. N is total number of participants and N case is for number of T2D patients. Source data are provided as a Source Data file.

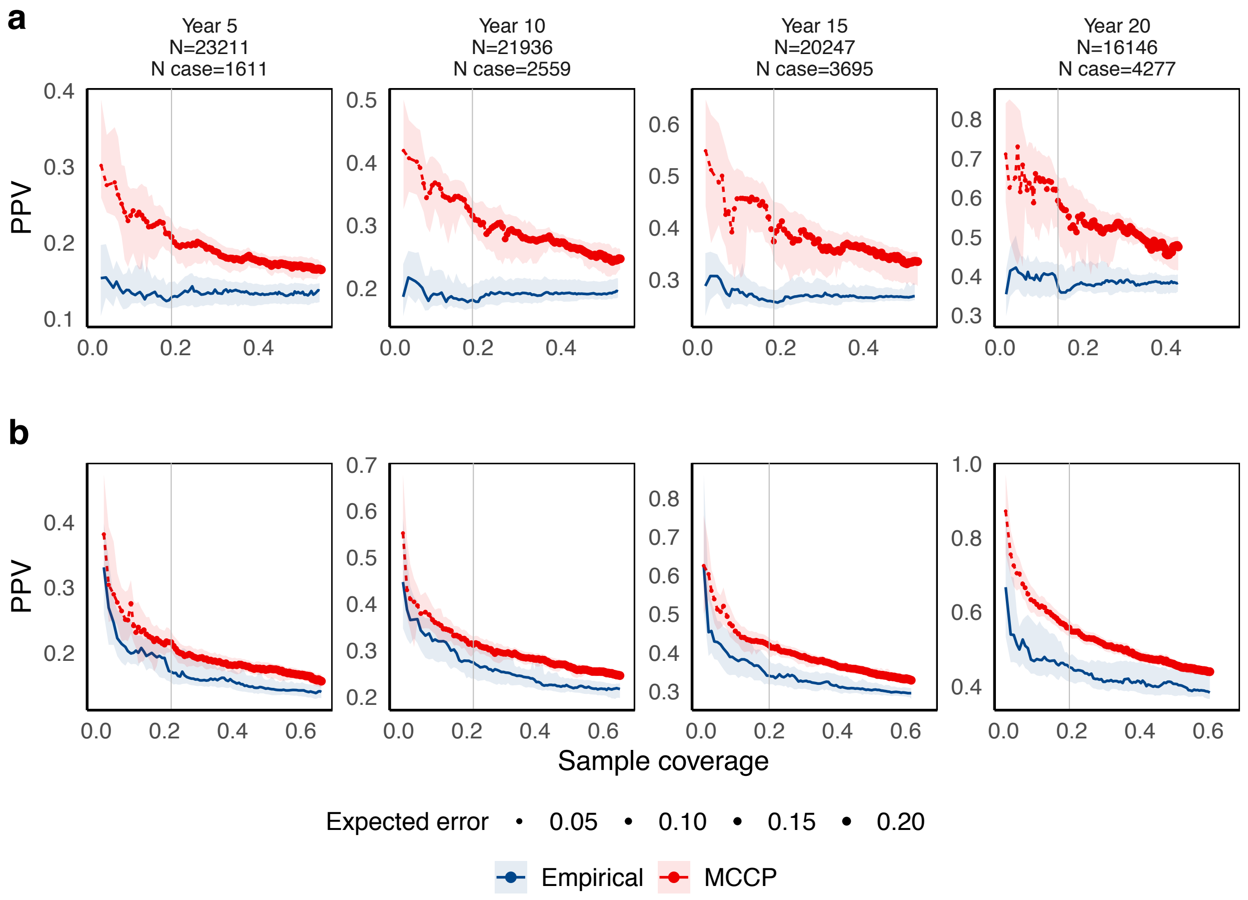

Supplemental Figure 11. Comparison of the performance of MCCP on T2D status in follow-ups (at year 5, 10, 15 and 20) with the empirical method using PRS and additional information (age, sex and PC1-6) in MCCP. a) NPVs in follow-up year 5, 10, 15 and 20, using models of MCCP and the empirical method, built at baseline. b) NPVs for MCCP and empirical method built at each time point. MCCP and empirical method are implemented at follow-up year 5, 10, 15 and 20, respectively. For MCCP, sample coverage (x axis) indicates the proportion of samples predicted as cases or controls, whereas, for the empirical method, it indicates extreme PRS, e.g., top and bottom 5% of PRS. The expected error rates for MCCP are indicated by the size of data points up to 0.20. Vertical lines correspond to an expected error of 0.05 from the MCCP. The solid lines and shades represent the median and 95% confidence intervals of NPVs. N is total number of participants and N case is for number of T2D patients. Source data are provided as a Source Data file.

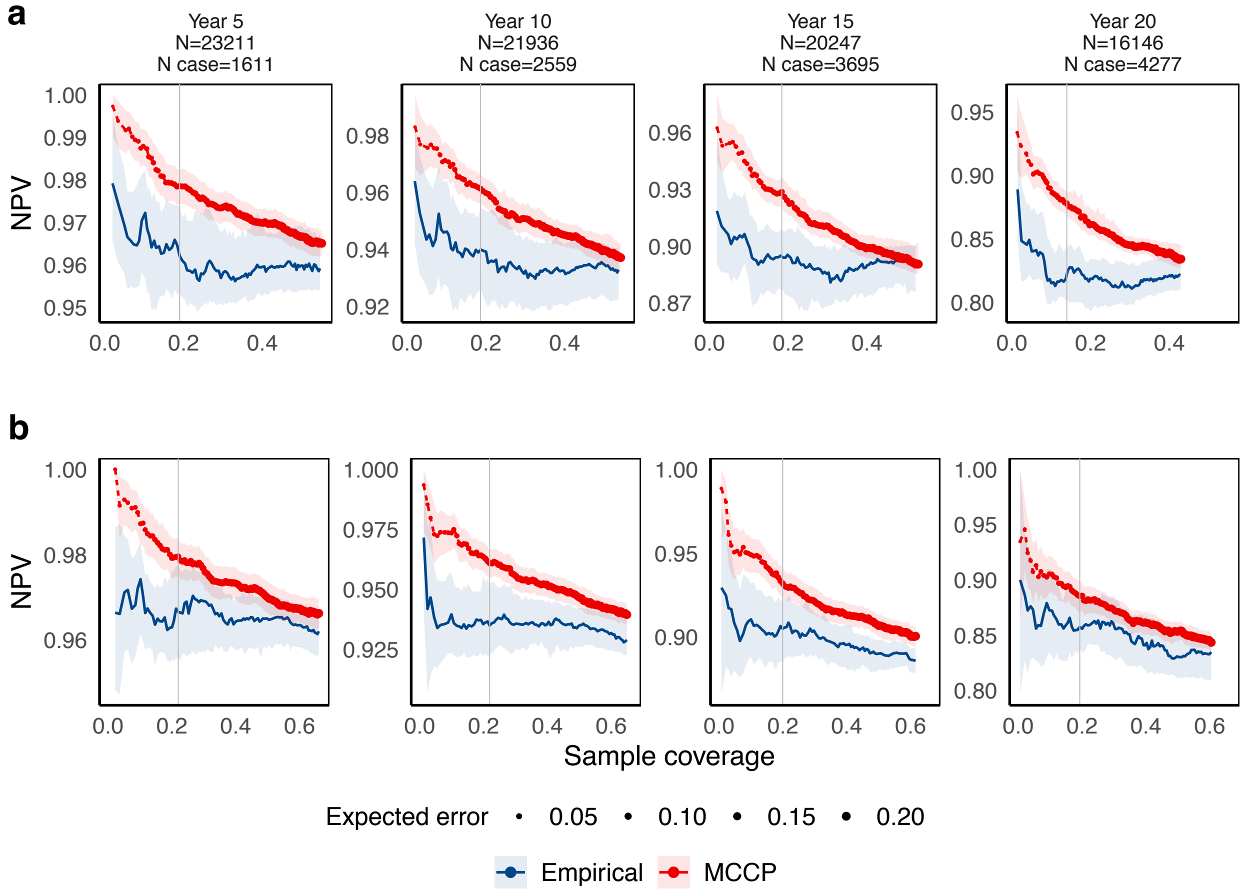

Supplementary Note

Regeneron Genetics Center Banner Author List and Contribution Statements

All authors/contributors are listed in alphabetical order.

RGC Management and Leadership Team
Goncalo Abecasis, Ph.D., Aris Baras, M.D., Michael Cantor, M.D., Giovanni Coppola, M.D., Aris Economides, Ph.D., Luca A. Lotta, M.D., Ph.D., John D. Overton, Ph.D., Jeffrey G. Reid, Ph.D., Alan Shuldiner, M.D.

Contribution: All authors contributed to securing funding, study design and oversight. All authors reviewed the final version of the manuscript.

Sequencing and Lab Operations
Christina Beechert, Caitlin Forsythe, M.S., Erin D. Fuller, Zhenhua Gu, M.S., Michael Lattari, Alexander Lopez, M.S., John D. Overton, Ph.D., Thomas D. Schleicher, M.S., Maria Sotiropoulos Padilla, M.S., Karina Toledo, Louis Widom, Sarah E. Wolf, M.S., Manasi Pradhan, M.S., Kia Manoochehri, Ricardo H. Ulloa.

Contribution: C.B., C.F., K.T., A.L., and J.D.O. performed and are responsible for sample genotyping. C.B, C.F., E.D.F., M.L., M.S.P., K.T., L.W., S.E.W., A.L., and J.D.O. performed and are responsible for exome sequencing. T.D.S., Z.G., A.L., and J.D.O. conceived and are responsible for laboratory automation. M.P., K.M., R.U., and J.D.O are responsible for sample tracking and the library information management system.

Genome Informatics
Xiaodong Bai, Ph.D., Suganthi Balasubramanian, Ph.D., Leland Barnard, Ph.D., Andrew Blumenfeld, Gisu Eom, Lukas Habegger, Ph.D., Young Hahn, Alicia Hawes, B.S., Shareef Khalid, Jeffrey G. Reid, Ph.D., Evan K. Maxwell, Ph.D., William Salerno, Ph.D., Jeffrey C. Staples, Ph.D., Ashish Yadav, M.S.

Contribution: X.B., A.H., W.S. and J.G.R. performed and are responsible for analysis needed to produce exome and genotype data. G.E., Y.H., and J.G.R. provided compute infrastructure development and operational support. S.K., S.B., and J.G.R. provide variant and gene annotations and their functional interpretation of variants. E.M., L.B., J.S., A.B., A.Y., L.H., J.G.R. conceived and are responsible for creating, developing, and deploying analysis platforms and computational methods for analyzing genomic data.

Research Program Management
Marcus B. Jones, Ph.D., Lyndon J. Mitnaul, Ph.D.

Contribution: All authors contributed to the management and coordination of all research activities, planning and execution. All authors contributed to the review process for the final version of the manuscript.
