# Supplementary material for: Translating polygenic risk scores for clinical use by estimating the confidence bounds of risk prediction
Source: Nat Commun. 2021 Sep 6;12:5276. doi: 10.1038/s41467-021-25014-7 (PMC8421428; doi:10.1038/s41467-021-25014-7)
Supplement: Supplementary file 2 — Reporting Summary [file 41467_2021_25014_MOESM2_ESM.pdf]

nature research

Corresponding author(s): Jiangming Sun, Kasper Lage

Last updated by author(s): Mar 31, 2021

Reporting Summary

Nature Research wishes to improve the reproducibility of the work that we publish. This form provides structure for consistency and transparency in reporting. For further information on Nature Research policies, see our Editorial Policies and the Editorial Policy Checklist.

Statistics

For all statistical analyses, confirm that the following items are present in the figure legend, table legend, main text, or Methods section.

n/a	Confirmed	
☐	☒	The exact sample size (*n*) for each experimental group/condition, given as a discrete number and unit of measurement
☒	☐	A statement on whether measurements were taken from distinct samples or whether the same sample was measured repeatedly
☐	☒	The statistical test(s) used AND whether they are one- or two-sided<br>*Only common tests should be described solely by name; describe more complex techniques in the Methods section.*
☐	☒	A description of all covariates tested
☐	☒	A description of any assumptions or corrections, such as tests of normality and adjustment for multiple comparisons
☐	☒	A full description of the statistical parameters including central tendency (e.g. means) or other basic estimates (e.g. regression coefficient) AND variation (e.g. standard deviation) or associated estimates of uncertainty (e.g. confidence intervals)
☐	☒	For null hypothesis testing, the test statistic (e.g. *F*, *t*, *r*) with confidence intervals, effect sizes, degrees of freedom and *P* value noted<br>*Give P values as exact values whenever suitable.*
☒	☐	For Bayesian analysis, information on the choice of priors and Markov chain Monte Carlo settings
☒	☐	For hierarchical and complex designs, identification of the appropriate level for tests and full reporting of outcomes
☐	☒	Estimates of effect sizes (e.g. Cohen's *d*, Pearson's *r*), indicating how they were calculated

Our web collection on statistics for biologists contains articles on many of the points above.

Software and code

Policy information about availability of computer code

Data collection	No software was used.
Data analysis	Scripts to perform central analyses described here are stored at https://github.com/sunjiangming/PRS_MCCP; additional software used includes: PLINK v1.90b5.2, KING 2.2.4, GCTA v1.25.2, R packages: glmnet_2.0-16, doParallel_1.0.14, foreach_1.4.4, caret_6.0-82, impute_1.56.0, rms_5.1-2, pROC 1.16.2, Zelig 5.1.7

For manuscripts utilizing custom algorithms or software that are central to the research but not yet described in published literature, software must be made available to editors and reviewers. We strongly encourage code deposition in a community repository (e.g. GitHub). See the Nature Research guidelines for submitting code & software for further information.

Data

Policy information about availability of data

All manuscripts must include a data availability statement. This statement should provide the following information, where applicable:

- Accession codes, unique identifiers, or web links for publicly available datasets
- A list of figures that have associated raw data
- A description of any restrictions on data availability

All GWAS summary statistics used in this study are publicly available in the following repositories: Coronary ARtery DIsease Genome wide Replication and Meta-analysis plus The Coronary Artery Disease Genetics consortium (coronary artery disease), http://www.cardiogramplusc4d.org/data-downloads/; DIAbetes Genetics Replication And Meta-analysis consortium (type 2 diabetes mellitus), https://diagram-consortium.org/downloads.html; International Inflammatory Bowel Disease Genetics Consortium (inflammatory bowel disease), https://www.ibdgenetics.org/downloads.html; Breast Cancer Association Consortium (breast cancer), http://bcac.ccge.medschl.cam.ac.uk/bcacdata/oncoarray/oncoarray-and-combined-summary-result/; Psychiatric Genomics Consortium (schizophrenia), https://www.med.unc.edu/pgc/download-results/scz/. Data from 1000 Genomes Project can be accessed at ftp://ftp.1000genomes.ebi.ac.uk/vol1/ftp/.

UK Biobank data are available to registered investigators upon approval via http://www.ukbiobank.ac.uk. Data from the MDC study can be applied for access through https://www.malmo-kohorter.lu.se. In accordance with the consent structure of iPSYCH and Danish law, individual level genotype and phenotype data from the iPSYCH study are not able to be shared publicly.

Field-specific reporting

Please select the one below that is the best fit for your research. If you are not sure, read the appropriate sections before making your selection.

☒ Life sciences ☐ Behavioural & social sciences ☐ Ecological, evolutionary & environmental sciences

For a reference copy of the document with all sections, see nature.com/documents/nr-reporting-summary-flat.pdf

Life sciences study design

All studies must disclose on these points even when the disclosure is negative.

Sample size	Sample size for simulation data is 10 000. In real data sets, all collected samples from respective study restricted to European unrelated population were used. All these data sets have large sample sizes more than 100 subjects - minimum number recommended for PRS analysis (Nat Protoc. 2020 Sep;15(9):2759-2772).<br>UK Biobank dataset, including 276,312 European unrelated participants, was used in this study.<br>We used data from the iPSYCH case-cohort study: A random, representative sample of the Danish neonatal biobank for individuals born between 1981 and 2005. All data was initially collected in 2012 and psychiatric diagnoses were later updated, complete through 2014. Data was ascertained according to a case-cohort design where the size of the cohort (random sample) was chosen to represent 2% of the broader population it was sampled from (population of Denmark born between 1981 and 2005). Cases were all cases in the population at the time of ascertainment.<br>We used data from the MDC study: A prospective cohort study including 30446 men and women (born 1923–1950) from the city of Malmö. The participants were examined between 1991 and 1996. T2D events have been followed up until 2016-12-31 using national and local hospital records and death certificates. Both prevalent and incident T2D cases at five time points (baseline, follow-up year 5, 10, 15 and 20) were included in the present study.
Data exclusions	For breast cancer, only 147,317 women from the UK Biobank were considered.<br>For SCZ, among the 78,050 samples with genotype data available in the iPSYCH case-cohort study, 5,353 were excluded according to genotype and imputation quality control procedures described in great detail in PMID:28924187 and PMID:30692689. These data exclusion criteria were determined before the study was designed or conducted.<br>For T2D, all cases that were specified as type 1, LADA, secondary diabetes or other were discarded from further analysis. Subjects without screening date at baseline or diabetic patients with first event of diabetes before the age of 40 years were also excluded.
Replication	We repeated the analysis across 6 datasets for 5 disorders , i.e., CAD, T2D, IBD, BRCA and SCZ, obtaining essentially similar performances in all cases.
Randomization	Samples were randomly split into k folds for analysis using createFolds() function in R package caret.
Blinding	We do not study interventions or other allocations across different groups. Therefore, blinding is not relevant in this study and was not employed.

Reporting for specific materials, systems and methods

We require information from authors about some types of materials, experimental systems and methods used in many studies. Here, indicate whether each material, system or method listed is relevant to your study. If you are not sure if a list item applies to your research, read the appropriate section before selecting a response.

Materials & experimental systems

n/a	Involved in the study	
☒	☐	Antibodies
☒	☐	Eukaryotic cell lines
☒	☐	Palaeontology and archaeology
☒	☐	Animals and other organisms
☐	☒	Human research participants
☒	☐	Clinical data
☒	☐	Dual use research of concern

Methods

n/a	Involved in the study	
☒	☐	ChIP-seq
☒	☐	Flow cytometry
☒	☐	MRI-based neuroimaging

Human research participants

Policy information about studies involving human research participants

Population characteristics	The UK Biobank are described in PMID: 25826379 and 30305743.<br>The detailed description of the iPSYCH cohort has been reported in PMID:28924187 and https://ipsych.dk.<br>Details of the MDC study and the recruitment are described in PMID:8429286 and https://www.malmo-kohorter.lu.se. Accessible upon application.
Recruitment	No original recruitment was performed in this study.
Ethics oversight	The iPSYCH study have been approved by Danish Scientific Ethics Committee, the Danish Health Data Authority, the Danish data protection agency and the Danish Neonatal Screening Biobank Steering Committee. This is in keeping with the strict ethical framework and the Danish legislation protecting the use of these samples. The MDC study was approved by the Ethics Committee of Lund University (LU 51–90).

Note that full information on the approval of the study protocol must also be provided in the manuscript.
